# A MOLECULAR LENS FOCUSED ON UNDERSTANDING ALZHEIMER'S DISEASE HEALTH DISPARITIES

**DOI:** 10.1093/geroni/igac059.219

**Published:** 2022-12-20

**Authors:** Jamaine Davis

**Affiliations:** Meharry Medical College, Nashville, Tennessee, United States

## Abstract

Alzheimer’s disease (AD), the most common cause of dementia in older adults, disproportionally affects African Americans (AA) with an incidence rate as much as three times higher, compared to other racial/ethnic groups. Multiple factors contribute to this racial disparity however, an in-depth understanding of the biological or genetic contributions does not exist. Compelling evidence indicate that genetic variants of the lipid transport protein, ABCA7, is more strongly associated with AD in African Americans. To understand how ABCA7 contributes to AD on the molecular level, we used a combination of structural and cell biology techniques. Our results suggest that the ABCA7 variant (T319A) reported to confer risk in AA, may contribute to AD by reducing the levels of phosphoinositol bisphosphate (PIP2), a phospholipid reported to be decreased in the AD brain. These results provide a framework for targeting mechanisms that increase PIP2 levels as an effective strategy mitigating AD disparities.

